# Chronic toxoplasmosis and sleepiness in obstructive sleep apnea: Is there a link?

**DOI:** 10.1371/journal.pone.0235463

**Published:** 2020-07-01

**Authors:** Céline Dard, Sébastien Bailly, Jean-Louis Pépin, Marie-Pierre Brenier-Pinchart, Hélène Fricker-Hidalgo, Marie Peeters, Hervé Pelloux, Renaud Tamisier

**Affiliations:** 1 Parasitology and Mycology Laboratory, CHU Grenoble Alpes, Grenoble, France; 2 Institute for Advanced Biosciences (IAB), Team Host-Pathogen Interactions and Immunity to Infection, INSERM U1209—CNRS UMR5309, University Grenoble Alpes, Grenoble, France; 3 Inserm, CHU Grenoble Alpes, HP2, University Grenoble Alpes, Grenoble, France; University of Rome Tor Vergata, ITALY

## Abstract

**Introduction:**

Sleepiness is the main clinical expression of obstructive sleep apnea (OSA) syndrome resulting from upper airway collapse. Recent studies have discussed the fact that the presence of *T*. *gondii* cysts in the brain and the resulting biochemical and immunological mechanisms could be linked to neurobehavioral disorders. The aim of the present study was to explore the potential impact of chronic toxoplasmosis on sleepiness and on obstructive sleep apnea (OSA) severity in OSA obese patients.

**Materials and methods:**

A case control study on obese patients screened for OSA was performed. According to the sleep disorder and matched based on gender, age and body mass index (BMI), two groups of obese patients were selected from our sample collection database. All patients were tested for toxoplasmosis serological status measuring anti-*Toxoplasma* IgG and IgM levels. Univariable and multivariable logistic regression models were performed to assess the impact of chronic toxoplasmosis on sleepiness and OSA severity.

**Results:**

107 obese patients suffering from OSA were included in the study (median age: 53.3 years Interquartile range (IQR): [41.9–59.9]; median BMI: 39.4 kg/m^2^ IQR: [35.5–44.1], apnea-hypopnea index = 27.5 events/h [10.7–49.9]). Chronic toxoplasmosis was present in 63.4% and 70.7% of patients with or without sleepiness (p = 0.48), respectively and was not associated either to sleepiness (OR: 0.76, 95% CI: [0.52; 2.33], p = 0.64) or OSA severity (OR = 1.75, 95% CI: [0.51; 5.98] p = 0.37).

**Conclusion:**

Although chronic *Toxoplasma* infection in immunocompetent humans has been associated to several behavioral disorders or pathologies in recent literature, we demonstrate here that chronic toxoplasmosis is not associated to sleepiness and to sleep apnea syndrome severity in obese patients suspected of sleep apnea syndrome.

## Introduction

Obstructive sleep apnea (OSA) syndrome is a chronic sleep-related breathing disorder characterized by recurrent episodes of pharyngeal collapses during sleep with daytime sleepiness as the main symptom. OSA is a significant and growing health concern as affecting up to 4% of the general population [1, 2]. Sleepiness has been shown to impair quality of life by affecting cognitive and psychological functions and is associated with an increased risk of near misses and vehicle accidents [3, 4]. Brain damages related to hypoxic insults and/or impaired cerebral vascularization have been incriminated as the main factor of sleepiness, but others pathophysiological pathways including existing or acquired central nervous system susceptibility could also play a role in the onset of sleepiness [5–7]. Counterintuitively, sleepiness is not directly correlated to OSA syndrome severity appreciated by the apnea hypopnea index (AHI) nor sleep fragmentation appreciated by the arousal index [7]. Thus, others potential factors need to be explored.

*Toxoplasma gondii* is an apicomplexan parasite with a worldwide prevalence of 30% that is believed to increase the risk of some psychiatric and neurological disorders in humans [8, 9]. After primary infection through consumption of raw or undercooked meat containing parasite tissue cysts or by contaminated vegetables with oocysts, *T*. *gondii* disseminates by the bloodstream to encyst in the brain and muscles, causing chronic toxoplasmosis in humans [10, 11]. *T*. *gondii* cysts are believed to persist lifelong as quasi-latent but still dynamic and replicating entities in the brain and muscles [12–14]. In rodent, toxoplasmosis infection induces behavioral modifications that are supposed to be the result from brain lesions (medial hypothalamic zone and associated forebrain structures). These behavioral modifications make mice vulnerable to feline ant therefore ensure its transmission back to the definitive host [15]. This support a strong hypothesis that the parasite is able to alter a very specific brain domain that relate to change in host behavior [16]. In humans, cerebral cysts have long been regarded as non-pathogen and harmless for hosts, causing a lifelong asymptomatic infection in immunocompetent patients. Within the past 15 years, many studies attempted to link *T*. *gondii* (chronic infection established by a positive *Toxoplasm*a IgG) with numerous psychiatric and neurological disorders—including schizophrenia, Alzheimer disease, epilepsy, mood disorders or brain cancers—and yielded conflicting results [17–24]. In the same vein, some studies have demonstrated the link between chronic toxoplasmosis and traffic accidents [25–30]. Etiopathogenesis is poorly known but *T*. *gondii* could alter neurotransmitter pathways with an increased dopamine level and a decreased tryptophan level in the brain and particularly in the amygdala and hippocampus [31, 32]. Recent studies have shown that cyst formation within neurons is controlled by the immune system and results in a basal inflammation in the brain [33]. Indeed, immunological mechanisms related to chronic infection in the brain may also be implied in various diseases related to the central nervous system [34].

The etiology and pathogenesis of obstructive sleep apnea (OSA) syndrome is not yet definitive, evidence shows that the dysfunction of pharyngeal nerve and the atonia of the muscle innervated by these nerve could play an important role in the progress of OSA syndrome [35]. Several of the classic neurotransmitters and neuromodulators have now been identified that contribute to neurochemical regulation of pharyngeal motor neuron activity and airway patency [36]. Since, the neurobiology of upper airway control particularly by hypoglossal motoneurons from hypoglossal nucleus in brainstem is discussed, and that *Toxoplasma* cysts are present in brainstem, the hypothesis is that cysts present during chronic toxoplasmosis may be involved in obstructive sleep apnea [37, 38]. The inflammatory environment, neurotransmitter-mediated changes or modification of microRNA homeostasis or others subtle brain dysregulations induce by the presence of Toxoplasma cysts could be involved in hypoglossal nucleus dysregulation [39, 40]. To this extent we may hypothesizes that the presence of *T*. *gondii* would modulates sleepiness susceptibility and/or neural function of central regulation of upper airway muscle during sleep.

Thus, regarding the fact that chronic toxoplasmosis has been supposed to be involved in central nervous system disorders in many studies without definitive and clear biological or physiopathological background, we tried to evaluate if chronic toxoplasmosis (infecting almost 30% of human beings) could be a cofactor of sleepiness.

The primary objectives of this case-control study were to assess, in obese patients, any relationship between chronic toxoplasmosis and sleepiness. The relationship between toxoplasmosis and OSA syndrome severity was assessed in the same population as a secondary objective.

## Materials and methods

### Study design

A total of 107 adult obese patients (BMI > 30 kg/m^2^) investigated for suspect severe OSA syndrome with different subjective daytime sleepiness (Epworth sleep scale) were included in the study. Sleepiness and OSA syndrome patients were compared to control subjects in a case control study design. Each patient with sleepiness was matched with one patient which was not and each severe OSA syndrome patient was matched with one non-severe OSA syndrome patient. Patients and controls were matched (1 case for 1 control) on sex, age (± 5 years) and BMI (± 3 kg/m^2^).

The effect of chronic toxoplasmosis on excessive daytime sleepiness was studied with a case control comparison of two clusters of obese exhibiting sleepiness (cases) or not (control) and tested for toxoplasmosis serological status. The effect of chronic toxoplasmosis on OSA syndrome severity was studied with a case control comparison of two clusters of obese exhibiting severe OSA (cases) or non-severe OSA (controls) and tested for toxoplasmosis serological status.

### Subjects

Both groups of subjects came from an obese database of subjects recruited by advertisement in newspapers or addressed to the sleep laboratory of Grenoble Alpes University hospital for suspicion of sleep disordered breathing. These were included in the process of screening from a previous clinical study run in our department regarding obesity hypoventilation syndrome [41]. The selected patients were screened but do not have obesity hypoventilation syndrome and thus were excluded from the study after screening visit. The database and biological sample library that was constituted from these patients. All patients provided at the time of their initial inclusion written informed consent allowing future usage of their biological samples and data. Clinical information was collected in HP2 database during patient’s visits through questioning, physical examinations, anthropometric data, polysomnography studies, Epworth Sleepiness Scale collected during every visit according to physician follow-up [42]. Obesity was defined by a BMI above 30 kg/m^2^, excessive daytime sleepiness by an Epworth scale of 11 or above. OSA syndrome was investigated for by an overnight polysomnography in the sleep laboratory and severe OSA syndrome was defined by an apnea hypopnea index (AHI) above 30.

Patients selected in the database were retrospectively analyzed for toxoplasmosis infection by toxoplasmosis serological analysis using sera stored in biobank (n°DC-2008-582) at -20°C. Co-morbidities factors (cardiovascular, respiratory and inflammatory factors) associated with the risk of sleepiness and OSA syndrome were considered. Cardiovascular factors were histories of cardiovascular disease, myocardial infarction, high blood pressure, smoking, stroke and dyslipidemia. Respiratory factors were histories of chronic lung disease, respiratory functions and blood-gas measurements. Inflammatory factors were high-sensitivity C-reactive protein and tumor necrosis factor-α. These factors were assessed either by examination and questioning, either by biological analyses. The study was approved by the university hospital ethics committee. All patients signed a written informed consent.

### Polysomnography (PSG) and measurement of sleep breathing disorders

An overnight PSG was performed for each patient in order to characterize and record abnormal respiratory events during sleep according to standard criteria [43, 44]. Continuous recordings were taken with electrode positions C3/A2-C4/A1-Cz/01 of the international 10–20 Electrode Placement System, eye movements, chin electromyogram and ECG with a modified V2 lead. Airflow was measured with nasal pressure, associated with naso-buccal thermistor signals. Respiration was monitored with uncalibrated inductance plethysmography. Oxygen saturation (SaO2) was measured using a pulse oximeter (Biox-Ohmeda 3700; Ohmeda; Liberty Corner, NJ). Sleep and respiratory events were recorded and scored manually according to standard criteria [AASM 2007] [45]. For polysomnography, airflow was measured with nasal pressure prongs together with the sum of oral and nasal thermistor signals. Respiratory effort was monitored using abdominal and thoracic bands. Oxygen saturation was measured using a pulse oximeter. An apnea was defined as the complete cessation of airflow for at least 10 seconds and hypopnea as a reduction of at least 50% in the nasal pressure signal or a decrease of between 30% and 50% associated with either oxygen desaturation of at least 3% or an EEG arousal [45], both lasting for at least 10 seconds. Apneas were classified as obstructive, central or mixed according to the presence or absence of respiratory efforts. The classification of hypopneas as obstructive or central was based on the thoraco-abdominal band signal and the shape of the respiratory nasal pressure curve (flow limited aspect or not). The apnea / hypopnea index (AHI) defined as the number of apneas and hypopneas per hour of sleep was calculated.

### Endothelial function data

Endothelial dysfunction was assessed by reactive hyperhemia with finger plethysmographic methodology (RH-PAT, i.e Reactive Hyperhemia Peripheral Arterial Tonometry) using Endo-PAT device (Itamar Medical Ltd, Caesarea, Israel) as previously described [46, 47]. RH-PAT index was calculated as the natural logarithm of the average amplitude of PAT signal after 90 to 120 second deflation divided by average amplitude.

### Blood pressure measurements

Clinical systolic and diastolic blood pressures were measured by mercury sphygmomanometry, after 5 min in a sitting position and on three separate occasions, according to the European Society of Hypertension/European Society of Cardiology (ESH-ESC) guidelines.

### Assessment of biological measures

After separation of serum and plasma from blood cells by centrifugation, aliquots of serum and plasma were frozen at -80°C for toxoplasmosis serological assays and biomarkers analyses.

#### Toxoplasmosis serological analyses

IgG and IgM titrations of anti-*T*.*gondii* were performed in sera using a quantitative Enzyme Linked Fluorescent Assay (ELFA) (Vidas^®^ Toxo IgG and Vidas^®^ Toxo IgM, bioMérieux, Marcy l'Etoile, France) [48, 49]. If IgG and/or IgM were positive, results were confirmed using another Microparticular Enzyme immune Assay (MEIA) (Architect^®^, Abbott, Chicago, USA). All tests were done in accordance with the manufacturer's guidelines. Antibody titers for IgG were quantitatively expressed in IU/mL whereas IgM were expressed as an index. The cut offs defined by Vidas^®^, bioMérieux manufacturer were: (i) IgG (IU/mL): negative < 4.0; 4.0 ≤ equivocal (grey zone)< 8; ≥8 positive; (ii) IgM (index): negative < 0.55; 0.55 ≤ equivocal (grey zone) < 0.65; ≥ 0.65 positive. The cut offs defined by Architect^®^, Abbott manufacturer were: (i) IgG (IU/mL): negative < 1.6; 1.6 ≤ equivocal (grey zone)< 3.0; ≥3.0 positive; (ii) IgM (index): negative < 0.50; 0.50 ≤ equivocal (grey zone) < 0.60; ≥ 0.60 positive. Chronic toxoplasmosis was considered when IgG were above the threshold of positivity with both methods.

#### Biomarkers

Heparinized plasma glucose, triglycerides, total and HDL-cholesterol concentrations in samples were determined using enzymatic methods and spectrophotometry (Modular 700, Roche Diagnostics, Meylan France). Low-density lipoprotein (LDL)-cholesterol was calculated by Fried-wald’s formula [Total cholesterol–(HDL cholesterol + triglycerides/5)]. Serum insulin was measured by immunoradiometric sandwich assay (Bis-Insulin IRMA^®^, CisBio international, Gif-Sur-Yvette, France). The homeostasis model assessment resistance (HOMA-RI) index was calculated by the following equation: insulin (μIU/mL) x glucose (mmol/L)/22.5. Serum high-sensitive C-reactive protein (hsCRP) level was measured using automated immunonephelometry (Behring Nephelometer II Analyzer^®^, Dade Behring, Germany). Leptin and TNFα were measured by commercially available multiplex beads immunoassays (Fluorokine MAP Multiplex Human Cytokine Panel and Obesity Panel, R&D Systems, Minneapolis, USA) and read by the Bioplex 200 array reader (Bio-Rad Laboratories, Hercules, CA, U.S.A.) which uses Luminex xMAPTM Technology (Luminex Corporation, Austin, TX, U.S.A.).

### Statistical analysis

A descriptive analysis of the patient’s characteristics was performed using median and interquartile range for quantitative data and frequencies and percent for qualitative data. The baseline characteristics of groups were compared by the means of chi-square test for qualitative data, and Mann-Whitney test for quantitative data. Missing values were imputed using multiple imputation method (MCMC algorithm) resulting in the creation of ten datasets. A univariate conditional logistic regression analysis was performed to select variable on the basis of a p-value threshold of 0.2. A multivariable conditional logistic regression model was performed, including selected variables and presence of a chronic toxoplasmosis. A stepwise selection method was performed to select the final model. Statistical analyses were performed using SAS v9.4 (SAS Institute Inc., Cary, NC, USA.). A p-value of <0.05 was considered as significant.

Primary objective was the impact of chronic infection to *T*. *gondii* on excessive daytime sleepiness which was defined by an Epworth scale greater than 10. Secondary objectives were to assess the association between chronic infection to *T*. *gondii* and 1) severe OSA syndrome defined by AHI greater than 30 and 2) on excessive daytime sleepiness for patients with OSA syndrome.

## Results

### Effect of chronic toxoplasmosis on sleepiness

**Fig 1:**
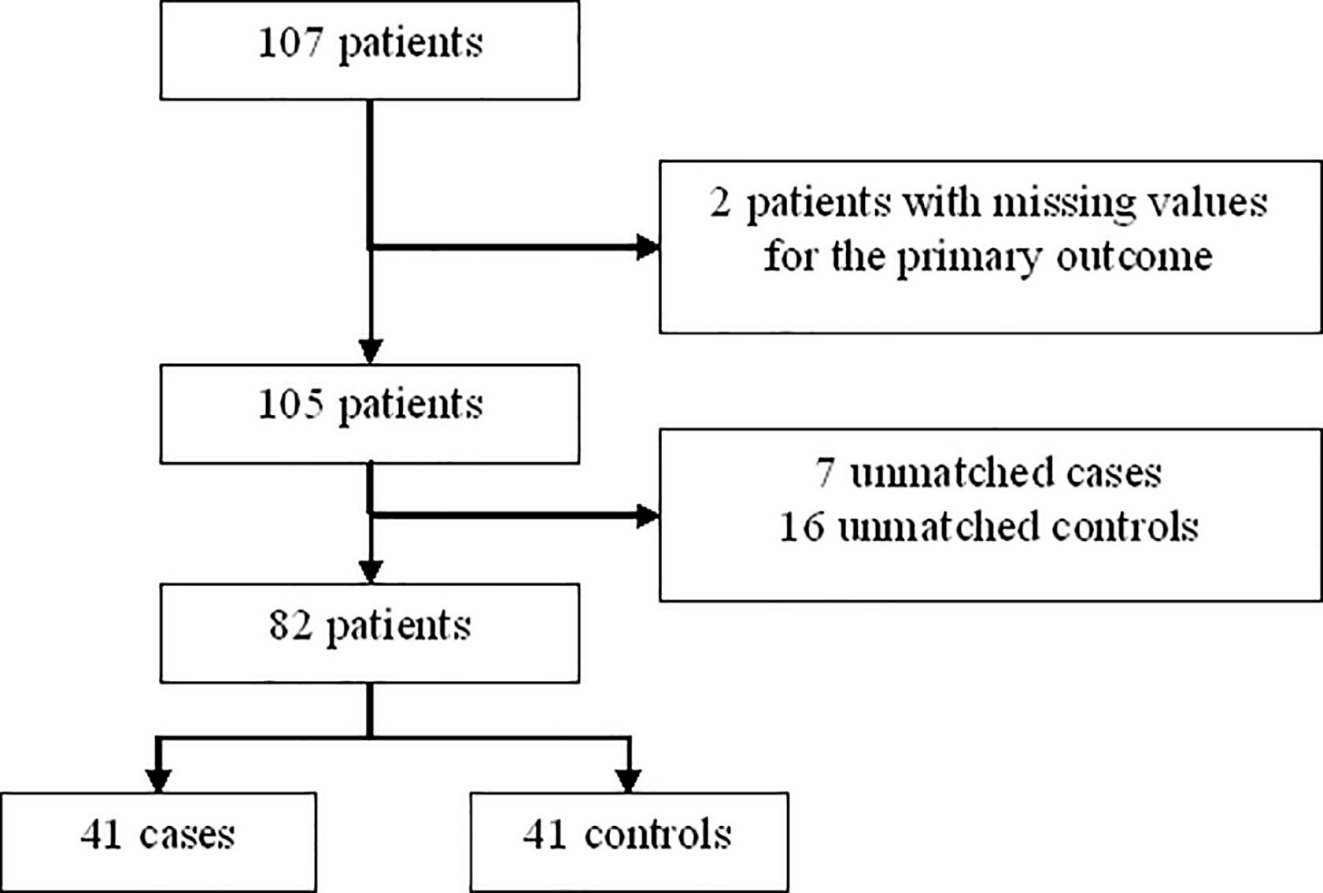
Flow chart of matching to study correlation between toxoplasmosis and effective daytime sleepiness (EDS).

A total of 107 patients were selected and 82 were included in the analysis with data on primary outcome (Fig 1). After matching on age, sex and BMI, 41 pairs were constituted by patients with sleepiness (cases) or not (controls). Patients had a median age of 53 years, (IQR: (41–60), were mainly female (62%) and had a high median BMI (39; IQR: 35–44) (Table 1). The only significant difference between patients with and without excessive daytime sleepiness was a higher proportion of patients with chronic respiratory disease in the group of patients with excessive daytime sleepiness (p = 0.03) (Table 1). The prevalence of a chronic toxoplasmosis was 70.7% and 63.4% in patients with excessive daytime sleepiness or without, respectively and was not significantly different between both groups (p = 0.48) (Table 1 and S1 Table). After univariable analysis, the following variables were included in the multivariate analysis: hypercholesterolemia, other history of chronic diseases, history of respiratory diseases, history of cardiovascular diseases, pH (as binary variable), and AHI as adjustment factor to assess the association between chronic toxoplasmosis and excessive daytime sleepiness. Only other history of chronic diseases was kept in the final multivariate model. Multivariable regression analysis showed no significant effect of chronic toxoplasmosis on sleepiness: OR = 0.76, 95% confidence interval: [0.52; 2.33], p = 0.64).

**Table 1:** Descriptive data of patient’s criteria for matching (case-control study).

	Without daytime sleepiness (n = 41)	Daytime sleepiness (n = 41)	p-value^a^
Age, median (IQR)	53.2 (41.3–59.9)	52.7 (42.7–58)	0.80
BMI, median (kg/m^2^, IQR)	40.1 (36.2–43.1)	39.1 (36.1–42.3)	0.55
Gender (male) (n, %)	14 (34.1)	17 (41.5)	0.49
**Apnea hypopnea index** (events/h)	22.5 (9–37.5)	30.4 (16–62)	0.14
Total sleep time	367.5 (321–404)	367.5 (334–390)	0.89
Time in stage I-II (min)	74 (62.8–79.9)	71.2 (62.2–80.9)	0.54
Time in stage III-IV (min)	4.9 (0–12.9)	2.5 (0–15.4)	0.95
Time in REM sleep (min)	21.7 (16.1–24.3)	21.5 (14.7–26.3)	0.64
TC90	3.7 (0.3–12)	7.9 (1.4–46)	0.11
**Cardiovascular disease (n, %)**	26 (63.4)	24 (58.5)	0.65
**Myocardial infarction (n, %)**	1 (2.5)	3 (7.5)	0.3
**High blood pressure (n, %)**	17 (42.5)	21 (52.5)	0.37
Systolic blood pressure (mmHg)	128 (120–139)	132 (123–146)	0.58
Diastolic blood pressure (mmHg)	74 (64–80)	73 (64–81)	0.99
Peripheral artery tonometry	2 (1.7–2.5)	2.1 (1.9–2.5)	0.85
**Smoking (n, %)**	15 (41.7)	21 (55.3)	0.24
**Stroke (n, %)**	1 (2.5)	2 (5)	0.56
**Hypercholesterolemia (n, %)**	13 (32.5)	6 (15)	0.07
Total-cholesterol (g.L^-1^)	2 (1.7–2.2)	2 (1.8–2.4)	0.91
LDL-cholesterol (g.L^-1^)	1.2 (0.9–1.3)	1.2 (1–1.4)	0.55
HDL-cholesterol (g.L^-1^)	0.5 (0.4–0.5)	0.4 (0.4–0.5)	0.69
Triglycerides (g.L^-1^)	1.4 (0.9–2)	1.5 (1–2.1)	0.34
**Diabetes (n, %)**	11 (27.5)	10 (25)	0.80
Glycemia (mmol/L)	5.6 (5–6.9)	5.9 (5.1–7.9)	0.43
Leptin (mmol/L)	25 (15–43)	21 (14–33)	0.32
HOMA-RI (mmol/L)	2.2 (1.7–3.6)	2.6 (1.4–5.1)	0.58
**Inflammatory state**			
hsCRP (mg/L)	4.8 (2.9–9)	4.5 (1.8–14)	0.98
TNF-α (ng/mL)	4 (2–10)	4 (2–8.6)	0.68
**Chronic lung disease (n, %)**	4 (10)	12 (30)	0.03
**Respiratory volumes**			
Forced expiratory volume in one second (FEV1)	2.8 (2.1–3.4)	2.6 (2.2–3.2)	0.66
Residual volume	2.5 (2.2–3)	2.4 (2.2–3.1)	0.98
Inspiratory capacity	2.9 (2.3–3.5)	2.8 (2.4–3.5)	0.89
Total lung capacity	6.3 (5.1–6.8)	5.9 (5–6.9)	0.67
Functional residual capacity	3.2 (2.7–3.5)	3 (2.6–3.6)	0.32
Forced vital capacity	3.7 (2.7–4.2)	3.1 (2.7–3.9)	0.62
HCO_3_ (mmol/L)	25.4 (23.9–27.4)	25.2 (24.3–26.6)	0.95
pH	7.4 (7.4–7.4)	7.4 (7.4–7.4)	0.3
PaO_2_ (kPa)	10.4 (9.9–11.4)	10.5 (10.1–11.1)	0.94
PaCO_2_ (kPa)	5.2 (5–5.6)	5.3 (5.1–5.6)	0.65
SaO_2_ (%)	0.96 (0.95–0.97)	0.96 (0.95–0.97)	0.49
**Chronic toxoplasmosis (n, %)**	29 (70.7)	26 (63.4)	0.48

Descriptive data related to sleep and ventilation during sleep, cardiovascular associated factors, inflammatory state, respiratory factors and repartition of patients with chronic toxoplasmosis among the two groups (excessive daytime sleepiness or not). Values are expressed in effective (frequency) for qualitative data and median (interquartile range) for quantitative data.

^a^P-value: chi-square or exact Fisher test for qualitative value, Mann-Whitney test for quantitative value.

BMI, body mass index; IQR, interquartile range; REM, rapid eye movement; TC90, time during which arterial O2 saturation was less than 90%; hsCRP, high-sensitivity C-reactive protein, TNF-α, tumor necrosis factor-α, HCO3 serum bicarbonates, PaO_2_ and PaCO_2_ for arterial partial pressure in oxygen and carbon dioxide respectively and SaO2 hemoglobin oxygen saturation.

### Effect of chronic toxoplasmosis on OSA syndrome severity

**Fig 2:**
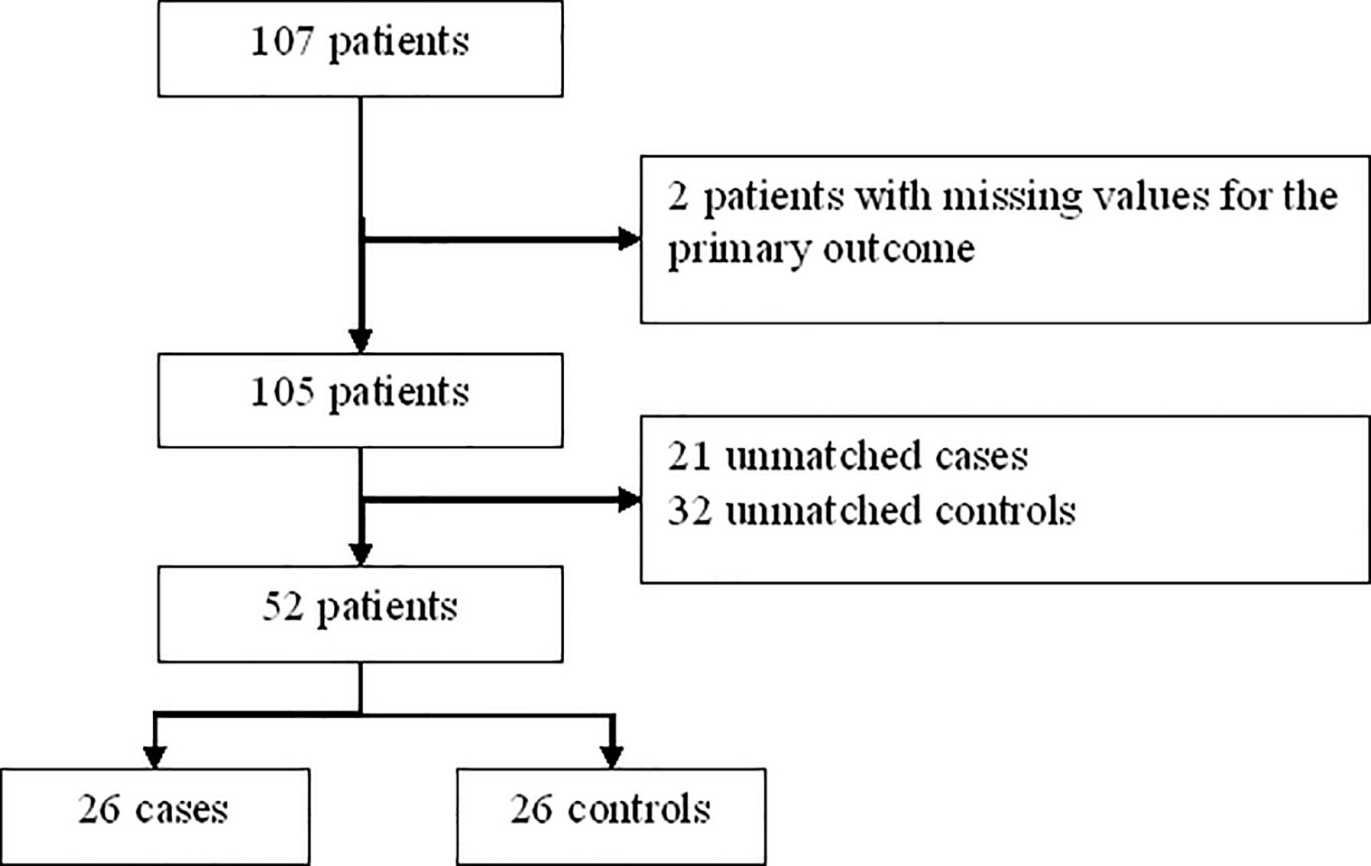
Flow chart of matching to study correlation between toxoplasmosis and OSA syndrome severity.

By considering patients with severe OSA syndrome, 26 cases (severe OSA, AHI>30) were matched to 26 controls (no and mild OSA) (Fig 2, Table 2 and S2 Table). By design there were significant differences between groups in apnea hypopnea index (AHI), time below 90% (TC90) and mean SpO2 during sleep. As could be expected the OSA severity impact on sleep macrostructure with a decrease in slow wave sleep in more severe patients. Only PaO2 of arterial blood gazes was different with a lower value in severe OSA (10.4 kPa [9.9; 11.0] *vs* 11.1 kPa [10.2; 11.9], p = 0.03).

**Table 2:** Descriptive data of no severe versus severe OSA patient’s.

	No severe OSA (n = 26)	Severe OSA (n = 26)	p-value^a^
Age, median (IQR)	52.2 (42.7–58.4)	54.2 (41.3–59.8)	0.64
BMI, median (kg/m^2^, IQR)	39.2 (35.3–45.3)	40 (35.2–43.7)	0.96
Gender (male) (n, %)	7 (26.9)	7 (26.9)	1
**Apnea hypopnea index** (events/h)	**14.3 (5.6–23.3)**	**46.9 (38–82.3)**	**< .01**
Total sleep time	367.5 (315–407)	363.5 (326.5–391)	0.41
Time in stage I-II (min)	**70 (62.8–74.5)**	**76.8 (66.8–88.6)**	**0.03**
Time in stage III-IV (min)	**9.3 (4.7–16.9)**	**0 (0–10.9)**	**0.02**
Time in REM sleep (min)	21.2 (16.7–24.9)	17.3 (10.2–23)	0.09
TC90	**1 (0.2–4.6)**	**19.3 (1.7–60)**	**0.02**
**Cardiovascular disease (n, %)**	17 (65.4)	18 (69.2)	0.77
**Myocardial infarction (n, %)**	1 (4)	1 (4)	1
**High blood pressure (n, %)**	12 (48)	15 (60)	0.39
Systolic blood pressure (mmHg)	124.3 (114–136)	130.5 (118–140)	0.28
Diastolic blood pressure (mmHg)	72.5 (64–81)	70 (60–76)	0.25
Peripheral artery tonometry	2.2 (1.8–2.5)	2.2 (1.9–2.4)	0.88
**Smoking (n, %)**	8 (34.8)	12 (52.2)	0.23
**Stroke (n, %)**	1 (4)	1 (4)	1
**Hypercholesterolemia (n, %)**	7 (28)	4 (16)	0.31
Total-cholesterol (g.L^-1^)	1.9 (1.7–2.2)	2 (1.7–2.4)	0.55
LDL-cholesterol (g.L^-1^)	1.2 (1–1.3)	1.2 (0.9–1.4)	0.85
HDL-cholesterol (g.L^-1^)	0.4 (0.4–0.5)	0.4 (0.3–0.5)	0.87
Triglycerides (g.L^-1^)	1.4 (0.8–2.1)	1.5 (1.1–2.1)	0.73
**Diabetes (n, %)**	8 (32)	8 (32)	1
Glycemia (mmol/L)	5.5 (5–6.4)	6.1 (5.1–8.7)	0.22
Leptin (mmol/L)	28 (14–50)	24 (14–34)	0.34
HOMA-RI (mmol/L)	1.9 (1.5–3.6)	2 (1.7–5.6)	0.42
**Inflammatory state**			
hsCRP (mg/L)	3.8 (2.9–9)	5.7 (3–14)	0.64
TNF-α (ng/mL)	4 (2–8.5)	3.5 (1.8–9)	0.43
**Chronic lung disease (n, %)**	7 (28)	7 (28)	1
**Respiratory volumes**			
Forced expiratory volume in one second (FEV1)	2.5 (2.2–3.1)	2.7 (2.1–3.4)	0.56
Residual volume	2.5 (2.1–3.1)	2.3 (1.9–2.8)	0.28
Inspiratory capacity	2.8 (2.4–3.5)	2.8 (2.1–3.4)	0.74
Total lung capacity	5.5 (4.9–6.5)	6 (4.8–6.6)	0.74
Forced vital capacity	3 (2.7–3.8)	3.1 (2.7–4.1)	0.55
HCO_3_ (mmol/L)	25.8 (24.2–26.9)	25.2 (24.2–26.6)	0.57
pH	7.4 (7.4–7.4)	7.4 (7.4–7.4)	0.15
PaO_2_ (kPa)	**11.1 (10.2–11.9)**	**10.4 (9.9–11)**	**0.03**
PaCO_2_ (kPa)	5.2 (5–5.5)	5.4 (5.2–5.6)	0.23
SaO_2_ (%)	**1 (1–1)**	**1 (1–1)**	**0.02**
**Chronic toxoplasmosis (n, %)**	14 (53.8)	17 (65.4)	0.4

Descriptive data related to sleep and ventilation during sleep, cardiovascular associated factors, inflammatory state, respiratory factors, and repartition of patients with chronic toxoplasmosis among the two groups (severe OSA or not). Values are expressed in effective (frequency) for qualitative data and median (interquartile range) for quantitative data.

^a^P-value: chi-square or exact Fisher test for qualitative value, Mann-Whitney test for quantitative value.

BMI, body mass index; IQR, interquartile range; REM, rapid eye movement; TC90, time during which arterial O2 saturation was less than 90%; hsCRP, high-sensitivity C-reactive protein; TNF-α, tumor necrosis factor-α HCO3 serum bicarbonates, PaO2 and PaCO2 for arterial partial pressure in oxygen and carbon dioxide respectively and SaO2 hemoglobin oxygen saturation.

There was no significant difference for chronic toxoplasmosis between groups of OSA severity (univariable OR = 1.75, 95%CI: [0.51; 5.98], p = 0.37). No variable was significantly associated with OSAS severity to be introduced in a multivariable analysis.

## Discussion

This study brings out new data in the fashionable thematic of “neurological diseases and chronic toxoplasmosis”. Herein, we provide an evaluation of the potential link between chronic toxoplasmosis and excessive daytime sleepiness in OSA patient as a primary outcome and between chronic toxoplasmosis and OSA syndrome severity in obese patients. In this case-control study of 107 patients, no significant link between chronic toxoplasmosis and sleepiness and OSA severity was demonstrated. Only the presence of chronic lung disease in medical history was associated with an increase in sleepiness. The most appropriate biological tools to answer the question “Is there a link between chronic toxoplasmosis and neurological disorders?” are serological tools within case-control studies. This study stands out for the quality of the enrolment of patients and the quality of serological analyses (two serological techniques and results interpreted by experts) [50]. Indeed, some of the studies showing a link between chronic toxoplasmosis and neurological troubles have low scientific evidence-based level and a real lack of high quality and these results must be confirmed by methodological relevant studies.

A prolific literature has been published showing the relationship between toxoplasmosis infection and change in rodent behavior making them more vulnerable to feline predators [16, 51, 52]. The fact that the parasite can change the behavior of its host has led to hypothesize a potential link between chronic toxoplasmosis infection and some human neurologic diseases. The most studied hypothesis is between schizophrenia and toxoplasmosis [53, 54]. To this extent there is component evidence that chronic inflammation due to *T*. *gondii* in patients with genetic susceptibility may develop neurologic disorders and specifically schizophrenia [55].

Human is dead-end host for *T*. *gondii* but like all intermediary hosts infected by the parasite have Toxoplasma cysts in brain. Interestingly there are some structural and functional changes in the brain upon chronic *Toxoplasma* infection. This may be a strong hypothesis that supports the link between *Toxoplasma* infection and neurologic symptoms [39]. We hypothesized that the presence of Toxoplasma cysts in brainstem could be involved both in sleepiness and upper airway muscle control, chronic toxoplasmosis infection may modulate OSA symptoms or severity. But our population analysis demonstrates that there is no relationship between these OSA features and chronic *T*. *gondii* infection.

Several limitations of this study need to be pointed out. Firstly, this study involves a cohort of obese patients, mainly female, and results cannot be largely extrapolated to all OSA patients, young OSA patients highly symptomatic. This preliminary survey could be enlarged to other categories of patients including non-obese patients, in which the mechanism of OSA in more linked to abnormalities of upper airways muscles control compared to obese patient. Secondly, the size of the cohort (n = 107) is relatively limited and the statistical power may be insufficient to unmask any effect of toxoplasmosis on the severity of OSA.

In conclusion, chronic toxoplasmosis in humans does not appear to be a risk factor of OSA severity and sleepiness-related OSA in obese patients. Despite a challenging hypothesis, we demonstrate that there is no relationship between well-defined *Toxoplasma* infection and sleepiness and OSA severity, contrarily to what has been attempted to be demonstrated for many other behaviors or pathologies.

## Supporting information

S1 Table Individual data of the 82 patients used for matching (case-control study). (XLSX)

S2 Table Individual data of the 52 patients with no severe and severe OSA. (XLSX)
